# LIPID AND LIPOPROTEIN LEVELS IN HIV-INFECTED ADULTS WITH SEPSIS COMPARED TO HEALTHY HIV- INFECTED CONTROLS

**DOI:** 10.21010/ajid.v14i2.1

**Published:** 2020-07-31

**Authors:** Faheem Seedat, Frederick Raal, Neil Martinson, Ebrahim Variava

**Affiliations:** 1 Division of Endocrinology and Metabolism, Department of Internal Medicine, Faculty of Health Sciences, University of Witwatersrand, South Africa; 2 Department of Internal Medicine, Klerksdorp Tshepong Hospital Complex, North West Province Department of Health, University of the Witwatersrand, South Africa; 3 Perinatal HIV Research Unit (PHRU), MRC Soweto Matlosana Collaborating Centre for HIV/AIDS and TB, University of the Witwatersrand, South Africa

**Keywords:** Lipid, Lipoprotein, HIV- infected, sepsis, infection

## Abstract

**Background:**

In acute sepsis, reduced lipid and lipoprotein levels occur in HIV negative patients, in particular, low high-density lipoprotein cholesterol (HDL–c) levels are inversely correlated with sepsis severity and increased mortality. However, due to the limited data describing lipid and lipoprotein levels in septic HIV–infected individuals we aimed to investigate the changes in this subgroup.

**Materials and Methods:**

A prospective cross–sectional observational study of HIV–infected patients comparing admitted HIV – infected patients with sepsis to healthy controls from the antiretroviral therapy (ART) clinic. Non fasting - lipograms, ART use, diagnosis of tuberculosis (TB), markers of infection, renal function and mortality outcome to 3 months post discharge were reviewed.

**Results:**

Total cholesterol (TC), low–density lipoprotein (LDL–c) and HDL-c were all significantly lower in the sepsis group (p < 0.001). HDL–c was significantly associated with a higher white cell count (p = 0.018), higher C– reactive protein (p = 0.036) and low serum albumin (p < 0.001). In those with active TB (55%) HDL–c was reduced even further (0.55 vs. 0.72mmol/L, p = 0.013). Acute kidney injury (p = 0.560) and mortality at discharge (p = 0.097) or 3 months follow up (p = 0.953) was not associated with reduced HDL–c.

**Conclusion:**

Septic HIV–infected patients had significantly reduced lipid and lipoprotein levels at admission. Of note however, a low HDL–c was associated with markers of infection and reductions in HDL–c was more marked in those with active TB.

## Introduction

Total cholesterol (TC) and triglycerides (TG) are water insoluble plasma lipid molecules, whilst low density lipoprotein cholesterol (LDL-c) and high-density lipoprotein cholesterol (HDL-c) are lipoproteins (Feingold 2000). Lipoproteins comprise a central lipid core complexed to an apolipoprotein. In patients with risk factors for cardiovascular disease LDL-c and HDL-c are the two lipoproteins most frequently measured, however their measured serum levels are seldom utilized in acute care (Feingold 2000). Despite this, there is interest in the role of lipids and, especially, lipoproteins in acute illness and particularly their role in sepsis (Feingold and Grunfeld 2000). Both lipid and lipoprotein levels decrease in those with sepsis and of all the lipoproteins the greatest inverse correlation with severity of sepsis appears to be reductions in HDL-c concentration (van Leeuwen *et al.*, 2003; Chien *et al.*, 2005; Tanaka *et al.*, 2017; Hsiao *et al.*, 2018). Indeed, marked reductions in HDL-c in sepsis are independently associated with increased mortality; a 13-fold increase in risk of overall mortality at 30 days in patients with sepsis is reported in those with HDL-c levels less than 0.52mmol/L (Chien *et al.*, 2005). Following recovery, levels of HDL-c increase (van Leeuwen *et al.*, 2003; Chien *et al.*, 2005; Barlage *et al.*, 2009).

In acute HIV infection, TC, TG, LDL-c and HDL-c levels initially decline with a low HDL–c being the commonest abnormality and a mean value of 0.77mmol/L is reported in antiretroviral therapy (ART) naive African HIV - infected adults (Njoroge *et al.*, 2017). However, with the introduction of ART all lipid and lipoprotein levels tend to increase (Fontas *et al.*, 2004; Dave *et al.*, 2016; Di Biagio *et al.*, 2016; Taramasso *et al.*, 2018).

Although the changes in lipid and lipoprotein levels in HIV-infected patients, both prior to and following ART initiation, are well described (Dave *et al.*, 2016), there is limited data describing these changes in acute illness in HIV- infected patients. In particular, there is little data examining whether the acute changes in TC, TG, LDL – c and HDL - c observed in septic HIV negative patients also occur in HIV - infected individuals. We therefore examined differences in TC, TG, LDL–c and HDL-c levels in septic HIV-infected adults and compared them to healthy HIV-infected controls.

## Materials and Methods

### Study setting

This prospective cross–sectional observational study included two groups of patients in the months October 2014 through June 2015, at the Tshepong Hospital, Klerksdorp, South Africa. At this facility patients are managed for a range of acute medical problems but, due to the significant burden of HIV and tuberculosis (TB) in the region, a large proportion of patients require admission for treatment of underlying opportunistic infections, with underlying active TB being the most common. The first group included septic HIV-infected patients admitted to the acute medical unit who were followed up until discharge and subsequently monthly, for three months. The second group comprised clinically well HIV-infected outpatients receiving ART (healthy controls) who were being followed up as part of routine care at the Tshepong hospital HIV outpatient clinic and enrolled during a single clinic visit. In both data sets: patients had to be aged 18 years or older and all consented to the use of their clinical records for research purposes. The Human Research Ethics Committee of the University of Witwatersrand, Johannesburg, South Africa, approved this study (Clearance Certificate Number: M140742).

### Study definitions and inclusion criteria

Amongst admitted HIV-infected inpatients, those that fulfilled the study definition of sepsis were identified and compared to the control group of clinically well HIV-infected individuals. Septic patients were defined as those admitted with any infection diagnosed by the treating internal medicine physician with the presence of both an elevated temperature greater than 38 degrees Celsius and admission C–reactive protein (CRP) > 10mg/dL (Povoa *et al.*, 2005, Vandijck *et al.*, 2007; Sproston and Ashworth 2018; Gyawali *et al.*, 2019).

Healthy controls were identified in the ART outpatient clinic and defined as patients requiring only their routine prescription for ART. Those with either constitutional symptoms suggestive of TB or in whom a clinician suspected an acute infection were excluded. Only those from each group who had results of a lipogram were included in this analysis. To avoid confounding from secondary dyslipidemia, patients in both groups with underlying diabetes, chronic kidney disease, hypothyroidism and known hyperlipidemia were excluded from the study, as were all those receiving lipid lowering therapy.

### Study procedures

From the clinical record, the prior history and duration of ART use and medical co–morbidities were abstracted. Patients were followed to discharge and the following were noted: whether or not a diagnosis of new TB disease was made during admission–, the discharge diagnosis and mortality outcome at discharge. Following discharge, mortality outcome over three months follow up was also recorded. Laboratory results of inpatient blood samples were also recorded, these included: the CD4 count, HIV viral load, a –complete blood count, randomly timed lipogram and serum biochemistry. All laboratory investigations were performed by the same laboratory, the public sector National Health Laboratory Service. The same parameters were abstracted from the control group of patients.

### Statistics

Descriptive statistics are reported as medians and interquartile ranges (IQRs) for continuous variables and for categorical variables as proportions with 95% confidence intervals (95% CI). We compared the sepsis patients to healthy HIV-infected controls, using the Chi–square test for categorical variables; Mann–Whitney–and Kruskall– Wallis tests for continuous variables were used. Due to the small sample size a multivariable regression analysis was not performed. Statistical analyses were performed using STATISTICA version 12 (StatSoft).

## Results

**Figure 1:**
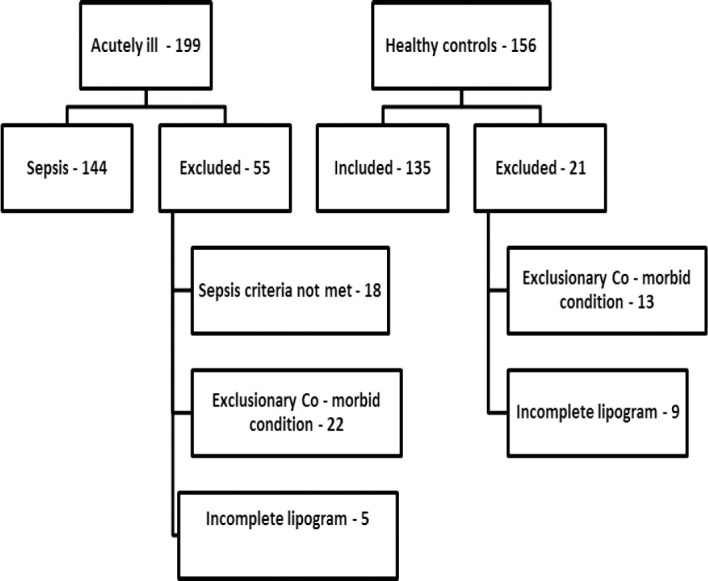
Study diagram showing of sepsis vs. healthy control groups.

Of the initial 199 patients in the septic group and 156 patients in the healthy HIV-infected control group, 55 and 21 patients were excluded, respectively (Figure 1); either they did not meet the study definition of sepsis, we identified the presence of an exclusionary co–morbid condition or the absence of complete lipogram results. Our final group was 144 sepsis and 135 healthy HIV-infected controls. The groups were well matched by age (median age of 39 [34–39] years old in both), gender (60% vs. 68% female) and body mass index (BMI) (20.8 [18.8–23.9] vs. 20.7 [17.6 – 24.7]kg/m^2^). 67% were receiving ART in the sepsis group and all were on ART in the HIV-infected healthy control group. Of those on ART, 48% in the sepsis group and all in the healthy control group were taking a tenofovir (TDF) - based regimen. Furthermore, the sepsis group had a smaller proportion of patients with viral suppression (38 vs. 52%) and a lower median CD4 count (100 [47 – 248] vs. 291 [92–501] cells/mm^3^). (Table 1).

**Table 1:** Baseline patient characteristics in the sepsis and healthy groups

	Sepsis (n = 144)	Healthy (n = 135)
Age (years), median(IQR)	39 (34 - 49)	39 (34 - 49)
Ethnicity n (%)		
Black	144 (99)	132 (98)
Mixed race	1 (1)	2 (1)
Indian	1 (1)	1 (1)
Gender (Female) n (%)	89 (60)	92 (68)
ART Use n (%)		
Naïve	48 (33)	0 (0)
TDF	69(48)	135 (100)
Other NRTI	27 (18)	0 (0)
NNRTI	92 (64)	114 (84)
PI	4 (3)	21 (16)
Newly Diagnosed TB n (%)	79 (55)	0 (0)
CD4 (cells/mm3), median (IQR)	100 (47 - 248)	291 (92 - 501)
VL < 1000 n (%)	54 (38)	70 (52)
Weight (kg), median (IQR)	56 (49 - 68)	55 (45 - 66)
BMI (kg/m2), median (IQR)	20.8 (18.8 - 23.9)	20.7 (17.6 - 24.7)
Creatinine (umol/L), median (IQR)	216 (156 - 482)	61 (50 - 74)
CRP (mg/L), median (IQR)	109 (48 - 192)	17 (5 - 64)
White Cell Count (x109/L), median (IQR)	8.59 (5.74 - 13.7)	5.6 (4.6 - 8.5)
Albumin (g/L), median (IQR)	18 (15 - 23)	35 (29 - 42)
Duration of stay in survivors n (%)		
< 10 days	64 (44)	
> 10 days	61 (41)	
Duration of stay in deceased n (%)		
< 10 days	6 (4)	
> 10 days	15 (10)	
Outcome n (%)		
Discharge		
Recovered	123 (85)	
Deceased	21 (15)	
3 Month follow up		
Recovered	95 (64)	
Deceased	38 (26)	
Unknown	15 (10)	

The leading infections in the septic patients included: TB (55%), gastroenteritis (30%), pneumonia (18%) and urinary tract infections (6%) and, of note, they had a significantly higher serum creatinine (SCr) concentration, white cell count (WCC) and CRP concentration and a lower serum albumin level than the healthy control group (p < 0.001).(Table 1).

**Figure 2:**
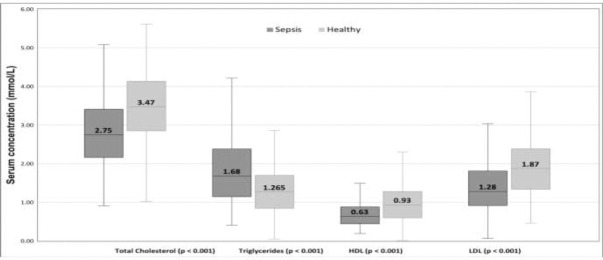
Comparison of serum lipids between the sepsis vs. the healthy group. Lipid levels (TC, LDL-c and HDL-c), amongst the sepsis group were all lower in those with TB compared to those without TB. However, only the HDL – c was significantly reduced (0.55 [0.42 – 0.73] vs. 0.72 [0.51 – 0.97]mmol/L) [p < 0.013] and correlated negatively RS = - 0.207 [p = 0.013] with the presence of newly diagnosed TB (Figure 3).

TC, LDL-c and HDL-c were lower in septic patients on admission than in healthy HIV-infected controls: (2.75 [2.16 – 3.41] vs. 3.47 [2.85 – 4.13]mmol/L; 1.29 [0.91 – 1.54] vs. 1.87 [1.33 – 2.38]mmol/L; 0.63 [0.44 – 0.88] vs. 0.93 [0.6 – 1.28]mmol/L) [p < 0.001], respectively. This was in contrast to TG levels which were raised in septic patients compared to healthy HIV-infected controls, but still within normal limits (1.68 [1.16 – 2.39] vs. 1.27 [0.86 – 1.72]mmol/L) [p < 0.001] (Figure 2).

**Figure 3:**
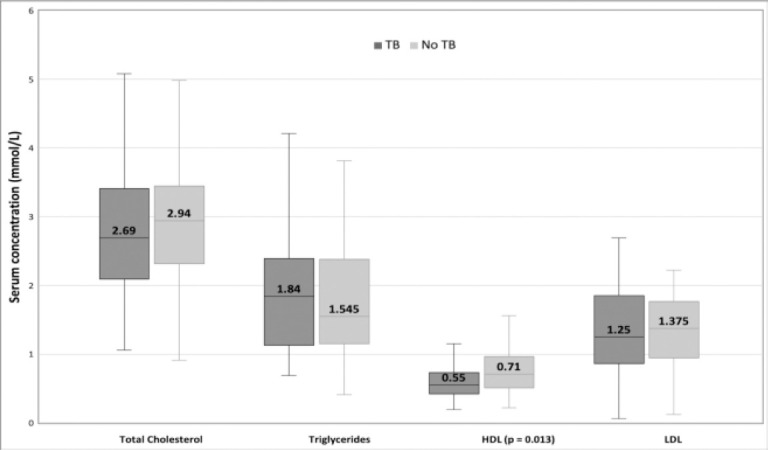
Serum lipids in septic patients compared by the presence of tuberculosis (TB). When the HDL – c levels in septic patients were studied in relation to infective markers: HDL -c was significantly lower in patients whose laboratory assays suggested an underlying infection or inflammatory process, these included: a WCC of more than 11 x 10^9^/L (p = 0.018), CRP greater than 30mg/L [p = 0.036] and serum albumin lower than 20g/dL [p < 0.001]. (Table 2).

**Table 2:** HDL concentrations categorised by HIV immune and virological status, markers renal function, infective markers and hospital stay and mortality outcome.

	HDL (median [IQR]) (mmol/L)	p - value
CD4 (cells/mm3)		
< 50	0.58 (0.39 - 0.71)	p = 0.042
50 – 350	0.62 (0.48 - 0.86)	
> 350	0.83 (0.51 - 1.22)	
HIV Viral Load < 1000 (copies/ml)		
< 1000	0.75 (0.55 - 1.09)	p = 0.001
> 1000	0.54 (0.4 - 0.72)	
Creatinine (umol/L)		
< 200	0.61 (0.43 - 0.88)	p = 0.560
> 200	0.65 (0.45 - 0.87)	
CKD - EPI (mL/min/1.73m2)		
< 30	0.63 (0.44 - 0.86)	p = 0.934
> 30	0.61 (0.44 - 0.88)	
AKI Severity		
Mild	0.64 (0.49 - 0.99)	p = 0.593
Moderate	0.58 (0.42 - 0.79)	
Severe	0.64 (0.44 - 0.84)	
CRP (mg/L)		
5 to 30	0.86 (0.52 - 1.16)	p = 0.036
Above 30	0.58 (0.44 - 0.76)	
WCC (x109/L)		
0 – 11	0.66 (0.50 -0.93)	p = 0.018
> 11	0.53 (0.35 - 0.74)	
Albumin (g/L)		
< 20	0.55 (0.41 - 0.72)	p < 0.001
> 20	0.77 (0.52 - 1.07)	
Duration of stay		
Survivors		
< 10 days	0.53 (0.4 - 0.86)	p = 0.034
> 10 days	0.66 (0.48 - 0.96)	
Deceased		
< 10 days	0.60 (0.5 - 0.73)	p = 0.205
> 10 days	0.71 (0.66 - 1.07)	
Outcome		
Discharge		
Recovered	0.58 (0.42 - 0.86)	p = 0.097
Deceased	0.7 (0.58 - 0.99)	
3 month follow up		
Recovered	0.65 (0.5 - 0.76)	p = 0.953
Deceased	0.66 (0.45 - 0.89)	

However, when the septic group was stratified by markers of acute kidney injury (AKI) (elevated SCr and reduced estimated glomerular filtration rate (eGFR) calculated by the chronic kidney disease epidemiology collaboration equation [CKD–EPI]) no significant difference in HDL–c measures were noted [p = 0.560 and p = 0.934, respectively]. Moreover, even when stratified by severity of AKI, according to the Kidney Disease Improving Global Outcomes criteria, there was no significant reduction in HDL–c amongst septic HIV infected patients [p = 0.593] (Khwaja 2012).

Despite high rates of mortality in the group with sepsis –15% in hospital and 24% at 3 months - reduced HDL – c levels were not associated with mortality either at discharge (0.58 vs 0.7mmol/L)[p = 0.097] or by the three month follow – up (0.65 vs. 0.66mmol/L)[p = 0.953]. In the deceased, TB was present in the vast majority both at discharge (71%) and at three months follow-up (71%).

Regarding other lipoproteins, LDL–c was significantly lower with a CRP greater than 90mg/L (1.125mmol/L [IQR: 0.81–1.52] vs. 1.395mmol/L [IQR: 0.94 – 1.82], [p = 0.03] but no other significant associations between markers of infection, AKI or mortality were noted for LDL -c, TC or TG.

## Discussion

In this study of HIV-infected adults, we compared those admitted to hospital with sepsis to a group of healthy controls. We describe significant reductions in HDL–c, LDL–c and TC but not TG levels in septic patients compared to the healthy control group. We report high rates of active TB (55%) and, of note, HDL–c was significantly lower in patients with newly diagnosed TB and in those where markers suggested infection or inflammation. However, no association was noted between HDL-c levels, AKI or mortality.

The lower levels of TC, LDL–c and HDL–c in HIV - infected patients with sepsis is similar to trends noted in septic HIV - negative patients where rapid decline in serum TC, LDL–c and HDL–c occurs at onset of sepsis, however TG changes are variable as noted by the opposing changes reported (Dunham *et al.*, 2003; van Leeuwen *et al.*, 2003; Chien et al. 2005; Tanaka *et al.*, 2017). Of all, it is a declining HDL–c, and its potential beneficial role in sepsis, that is best described (Morin *et al.*, 2015). Amongst septic HIV negative individuals it is proposed that the lipid rich walls of gram negative (lipopolysaccharides) [LPS] and gram positive (lipoteichoic acid) [LTA] bacteria are neutralised by HDL–c and cleared by reverse cholesterol transport with resultant low HDL-c concentrations (Morin *et al.*, 2015). Furthermore, HDL–c attenuates the immune response from both macrophages and endothelial cells through binding of LPS (Morin *et al.*, 2015). However, reduced levels of apolipoprotein A, due to diminished secretion, also occur in sepsis and may contribute to noted HDL-c reductions (van Leeuwen *et al.*, 2003). Reduced LDL–c levels are postulated to occur due to clearance of bacterial toxins by LDL–c. This occurs through LDL–c first binding to LPS which are then subsequently cleared by hepatic LDL–c receptors (Khovidhunkit *et al.*, 2004; Guirgis *et al.*, 2018). The reduced levels of LDL–c and HDL–c we note suggest that similar mechanisms, amongst HIV negative patients, resulting in reduced LDL–c and HDL–c may occur in HIV-infected individuals with acute sepsis, however, further clarification of these mechanisms are necessary. Elevations in TG we note are similarly noted in other studies, TG increases are postulated to occur due to increased hepatic very low density lipoprotein (VLDL) production stimulated by pro–inflammatory cytokines which are increased in sepsis (Khovidhunkit *et al.*, 2004).

In HIV negative patients the acute decrease in HDL–c during sepsis is negatively correlated with a rise in inflammatory cytokines: tumour necrosis factor–alpha, interleukin 6 and interleukin 10. Conversely, a positive correlation between declining HDL – c and serum albumin is noted (Chien *et al.*, 2005; Vavrova *et al.*, 2016). Likewise, in septic HIV-infected patients we observe similar relationships between HDL–c and CRP–a surrogate marker for inflammatory cytokines (Oberhoffer *et al.*, 1999)–and albumin. This is further reinforced by the lower HDL–c observed at higher WCCs. Intriguingly, the role of granulocyte-monocyte colony-stimulating factor (GM-CSF) has been investigated as a potential cholesterol lowering agent (Nimer *et al.*, 1988). Whether, increased production of GM–CSF to aid the production of white cells in sepsis may subsequently contribute to the acute drop in HDL–c remains unknown and is a possible area of further study.

In the patients newly diagnosed with TB (55%) we noted even greater reductions in HDL–c. There is limited data reporting on changes in plasma lipid and lipoproteins in active TB where initial reductions in TC, TG, LDL–c and HDL-c levels and subsequent recovery of lipid and lipoprotein levels following successful treatment occur (Taparia, Yadav et al. 2014). In the only reported study examining lipid profiles in HIV-infected patients with active TB from Ethiopia, HIV-infected patients had significantly lower TC, TG, LDL–c and HDL-c compared to the HIV negative groups(Gebremicael *et al.*, 2017). To our knowledge, no other study has compared lipid or lipoprotein levels in HIV-infected patients with newly diagnosed TB to TB negative HIV-infected controls. Regarding the role of cholesterol in TB there is inconsistency. Concern exists that cholesterol may be an energy source for *Mycobacterium tuberculosis* (MTB), however, cholesterol–rich diets have, in contrast, accelerated bacteriologic sterilization of MTB (Martens *et al.*, 2008; Akpovi *et al.*, 2013). It is suggested that, following TB infection, macrophage activation, free radical release and lipid peroxidation reduces lipid and lipoprotein levels (Gebremicael *et al.*, 2017). Interestingly, if compared to the LPS and LTA bacterial walls, MTB too has a lipid rich wall as lipid mycolic acids comprise 60% of the cell wall and other lipid constituents include: lipoarabinomannan, isoprenoid lipids and glycerophospholipids (Jackson 2014, Ghazaei 2018). We postulate that the reduced HDL-c level in TB patients may result from elimination of the MTB cell wall via reverse cholesterol transport – similar to gram negative and positive bacteria, however, further research is necessary to elucidate this mechanism(Morin *et al.*, 2015).

TDF is noted to have an intrinsic lipid lowering effect (Santos, Saumoy et al. 2015). Of interest, despite all patients in the healthy group using TDF compared to less than half amongst the septic group using TDF, we still note significant reductions in TC, LDL–c and HDL–c amongst the HIV–infected septic group. Suggesting, that the lipid lowering effect in the sepsis group should not be attributed to TDF. However, this is tempered by the lower CD4 count and fewer virally suppressed patients in the sepsis group which may have contributed to the lower lipid levels observed in the septic compared to healthy group (Floris-Moore *et al.*, 2006). Although a low CD4 count is shown to reduce HDL-c a South African study showed no association between reductions in TC or LDL–c and CD4 count (Dave *et al.*, 2016). As such, reductions in TC or LDL–c and CD4 count may still be attributed to alterations in lipid metabolism in acute sepsis.

Our study is limited by a small sample size and a second lipogram following recovery from sepsis to demonstrate improvement in lipid and lipoprotein levels is lacking. The use of a non–fasting lipogram is a limitation but the practicalities of obtaining a fasting lipogram amongst acutely ill patients precluded this. Furthermore, we did not measure specific inflammatory cytokine levels. The absence of critically ill HIV positive control group without sepsis is a shortcoming of the study. Whilst, differences between the two groups with regards immunological status, viral load and TDF use may serve as confounders when comparing lipid results between the two groups. The greater use of protease inhibitors, which also elevate lipid levels (Melroe, Kopaczewski et al. 1999), may have also contributed to elevated lipids in the healthy groups. However, the difference in prevalence of PI use between the groups was not large. Furthermore, the small sample size of this study did not allow for the analysis of initial lipid results as markers of prognosis and outcome.

## Conclusion

In septic HIV-infected patients, we note: TC, LDL–c and HDL–c are significantly lower than well HIV– infected controls and are similar to those described in septic HIV negative patients, however, of note these changes were more marked amongst those with active TB–in particular reductions in HDL-c. Further studies are necessary to determine the use of HDL-c as a prognostic indicator in acute sepsis amongst those with HIV; to clarify its potential immunomodulatory mechanisms; and the potential therapeutic implications of HDL-c modification that could improve outcomes in acute sepsis and MTB infection.

**List of abbreviations:**
AKI: - Acute kidney injury
ART: - Antiretroviral therapy
BMI: - Body mass index
CRP: - C – reactive protein
CI: - Confidence intervals
CKD – EPI: - Chronic kidney disease epidemiology collaboration equation
eGFR: - estimated glomerular filtration rate
GM – CSF: - Granulocyte - monocyte colony - stimulating factor
HDL – c: - High-density lipoprotein cholesterol
IQR: - Interquartile ranges
LPS: - Lipopolysaccharides, Lipoteichoic acid – LTA
LDL – c: - Low density lipoprotein
MTB: - *Mycobacterium tuberculosis*
SCr: - Serum creatinine
TC: - Total cholesterol
TG: - Triglycerides
TB: – Tuberculosis
VLDL: – very low density lipoprotein
WCC: - White cell count
